# 

**Published:** 1888-11-10

**Authors:** 


CONTENTS.

**City Magnates and their Work** - - - - 81

**A German Taptoo** - - - - - - - 82

**Hospital Finance and Economy.**—III - - - 83

**Within the Wards.**—A Berlin Physician on the Treatment of Consumption.—A Dangerous Lunatic - - - - 84

**The Doctor's Pulpit.**—The Seven Ages of Man—VI. - 85

**Turning Over New Leaves.**—"Self-Help a Hundred Years Ago" - - - - - - - - - 86

**Voyages in Vacation.**—VII. Copenhagen and the Baltic - 87

**Notes and News** - - - - - - - 88

**Everybody's Page** - - - - - - - 90

**Annotations.**—"False Doctoring."—The Flock or the Fleece ?—An Incompatible Mixture - - - - - - 91

**Little Men** - - - - - - - - 92

**False Impressions.** By Caroline Fothergill, Author of "An Enthusiast," "A Voice in the Wilderness," etc. - - 93

**The Student's Column.**—Examination Questions - - 94

**Miss M'Kellar and the Hospitals** - - - - 95

**Editor's Letter Box.**—Homeless.—A Case for Help; etc - 95

**Scraps and Gleanings—Amusements and Relaxation** 96

**EXTRA SUPPLEMENT.—THE NURSING MIRROR.**

**En Passant**—The Matron of Loughton.—Church Army Nurses.—Jubilee Nurses in Scotland.—A Suggestion.—Nurses in Vienna.—French Sisters of Charity.—The March of Progress - - - - - - - - - xxi
**Original Articles.**—Loquacity - - - - - - xxii
**Everybody's Opinion.**—National Pension Fund for Nurses.—Nurses and Presents.—Home for Nurses - - - xxiii
**Appointments** - - - - - - - - xxiii
**Vacancies** - - - - - - - - - xxiii
**Notes and Queries** - - - - - - - xxiii

